# The effects of cerebrospinal fluid tap-test on idiopathic normal pressure hydrocephalus: an inertial sensors based assessment

**DOI:** 10.1186/s12984-019-0638-1

**Published:** 2020-01-16

**Authors:** Alberto Ferrari, David Milletti, Giulia Giannini, Sabina Cevoli, Federico Oppi, Giorgio Palandri, Luca Albini-Riccioli, Paolo Mantovani, Laura Anderlucci, Pietro Cortelli, Lorenzo Chiari

**Affiliations:** 1 Health Sciences and Technologies - Interdepartmental Center for Industrial Research (CIRI-SDV), Alma Mater Studiorum - University of Bologna, Bologna, Italy; 2 Unit of Rehabilitation Medicine, IRCCS Istituto delle Scienze Neurologiche di Bologna, Bologna, Italy; 3 Unit of Neurology, IRCCS Istituto delle Scienze Neurologiche di Bologna, Bologna, Italy; 4 Department of Biomedical and Neuromotor Sciences (DIBINEM), University of Bologna, Bologna, Italy; 5 Unit of Neurosurgery, IRCCS Istituto delle Scienze Neurologiche di Bologna, Bologna, Italy; 6 Unit of Neuroradiology, IRCCS Istituto delle Scienze Neurologiche di Bologna, Bologna, Italy; 7 Department of Statistical Sciences, University of Bologna, Bologna, Italy; 8 Department of Electrical, Electronic, and Information Engineering “Guglielmo Marconi” (DEI), University of Bologna, Bologna, Italy

**Keywords:** Idiopathic normal pressure hydrocephalus, Inertial measurement units, Gait analysis, TUG test, CSF tap test

## Abstract

**Background:**

Gait disturbances are typical of persons with idiopathic normal pressure hydrocephalus (iNPH) without signs distinctive from other neurodegenerative and vascular conditions. Cerebrospinal fluid tap-test (CSF-TT) is expected to improve the motor performance of iNPH patients and is a prognostic indicator in their surgical management. This observational prospective study aims to determine which spatio-temporal gait parameter(s), measured during instrumented motor tests, and clinical scale(s) may provide a relevant contribution in the evaluation of motor performance pre vs. post CSF-TT on iNPH patients with and without important vascular encephalopathy.

**Methods:**

Seventy-six patients (20 with an associated vascular encephalopathy) were assessed before, and 24 and 72 h after the CSF-TT by a timed up and go test (TUG) and an 18 m walking test (18 mW) instrumented using inertial sensors. Tinetti Gait, Tinetti Balance, Gait Status Scale, and Grading Scale were fulfilled before and 72 h after the CSF-TT. Stride length, cadence and total time were selected as the outcome measures. Statistical models with mixed effects were implemented to determine the relevant contribution to response variables of each quantitative gait parameter and clinical scales.

**Results and conclusion:**

From baseline to 72 h post CSF-TT patients improved significantly by increasing cadence in 18 mW and TUG (on average of 1.7 and 2.4 strides/min respectively) and stride length in 18 mW (on average of 3.1 cm). A significant reduction of gait apraxia was reflected by modifications in double support duration and in coordination index.

Tinetti Gait, Tinetti Balance and Gait Status Scale were able to explain part of the variability of response variables not covered by instrumental data, especially in TUG. Grading Scale revealed the highest affinity with TUG total time and cadence when considering clinical scales alone.

Patients with iNPH and an associated vascular encephalopathy showed worst performances compared to pure iNPH but without statistical significance. Gait improvement following CSF-TT was comparable in the two groups. Overall these results suggest that, in order to augment CSF-TT accuracy, is key to assess the gait pattern by analyzing the main spatio-temporal parameters and set post evaluation at 72 h.

**Trial registration:**

Approved by ethics committee: CE 14131 23/02/2015.

## Background

Idiopathic normal pressure hydrocephalus (iNPH) is a syndrome characterized by chronic ventricular dilation, normal cerebrospinal fluid (CSF) pressure and the triad of symptoms: gait apraxia, urinary incontinence, and cognitive deficits [1]. Treating iNPH with the insertion of a ventricular peritoneal shunt allows draining excessive cerebrospinal fluid, [2] which in turn can lead up to a complete recovery [2, 3]. A recent systematic review reported symptoms improvement following shunt insertion in 71% of the three thousand patients analyzed in 64 published studies [3].

Still, the diagnosis of iNPH is complicated due to the considerable variability in its clinical presentation and course. Following current consensus guidelines, iNPH should be diagnosed from clinical history, physical examination, and brain imaging [2, 4, 5]. Practically, it is usually identified by the exclusion of other conditions once specific treatments for Parkinsonism or musculoskeletal diseases are ineffective. Nonetheless, a delayed diagnosis causes a disease progression to a point where treatment may be no longer effective [6].

The gait of iNPH patients is usually described as bradykinetic, shuffling, unstable on turning and with reduced walking speed [4]. Besides, it is typically magnetic, with an increased double support duration witnessing a difficulty in limb rising and step initiation [7]. Overall gait is apraxic, and this is usually the most disabling symptom and first sign onset, whereas cognitive impairment and urinary incontinence might appear in a later stage [4]. Gait apraxia is a common symptom also in patients affected by vascular encephalopathy [8]. Thus, iNPH and vascular encephalopathy conditions are often confused with each other. Moreover, vascular encephalopathy might result as a comorbidity of iNPH, potentially limiting the effectiveness of surgical treatment.

Supplementary prognostic tests, e.g., CSF tap-test (CSF-TT), have been used to attain a higher specificity and sensitivity for diagnosing iNPH and for predicting shunt response [2, 9]. The rationale is that temporary symptoms improvement from CSF drainage is prognostic for following shunt insertion. In a recent review study from Mihalj et al., the accuracy of CSF-TT in screening patients for shunting is 62%, but its negative predictive value is merely 37% [10]. This latter unsatisfactory result can be potentially ascribed to the use of ineffective tools in the analysis of post-CSF-TT improvements. In fact, the measure of gait changes is frequently based on qualitative, examiner-based evaluations rather than quantitative analyses, possibly leading to a misinterpretation of the findings [11]. Gait is commonly evaluated with the 10-m walking test (10 mW) having the test duration as a single outcome, or by clinical scales such as the iNPH Grading Scale [12]. This latter, in particular, is a clinician-rated scale aimed at separately assessing the severity of each of the three main symptoms [13]. As well, the Gait Status Scale was explicitly designed for analyzing gait disturbances of iNPH patients on the base of simple clinical observations [13].

Instrumented gait analysis may conversely be a crucial tool to augment CSF-TT accuracy allowing to objectively assess the improvements through a precise measure of the gait pattern [7]. Among different systems available for gait analysis, inertial measurement units (IMUs) are nowadays opening new perspectives in instrumenting motor tests [14]. The latest generation of IMUs are indeed wireless, small, lightweight, cost-effective and operating in real-time, therefore allowing the plug-and-play execution of gait analysis tests in the times of clinical routine [15]. Besides, the use of apps for mobile devices allows us to run the tests in any environment and to have detailed reports right after their executions [15, 16].

Just a few studies investigated the effects of CSF-TT through an instrumented gait analysis. Among these, Stolze et al. in a study involving 10 iNPH patients, reported gait speed and stride length as the most responsive parameters in a pre- vs. 24 h post-CSF-TT comparison, whereas cadence and balance remained unaffected [17]. More recently, Panciani et al. compared gait performances on 52 iNPH patients pre vs. few hours post-CSF-TT finding improvements in gait speed, stride length and double support duration [18]. Another study compared performances on a timed-up and go test (TUG) pre vs. 24 h post-CSF-TT revealing significant improvements in the sit-to-stand transition, walking time, and the number of steps employed to turn [19]. Allali et al. found a significant improvement of gait speed in single and dual tasking of 10 mW in a pre vs. 24 h post-CSF-TT [20]. In a study involving 74 patients, significant differences pre and 2-4 h post-CSF-TT were revealed for the Performance Oriented Mobility Assessment (Tinetti, [21]) and the Berg Balance Scale. Partially in contrast with these findings, TUG and 10 mW revealed improvements just in those patients assessed as eligible for shunt neurosurgery [22].

Gait changes after CSF-TT is a transient phenomenon, but literature does not offer an agreement about when it is the time point where the maximal modification can be expected [10]. Several studies analyzed the motor performances of patients not after a predefined amount of time, but on different days [23]. Other studies provided evidence of improvement within the first 24 h [7, 11, 18, 22]. In particular, Virhammar et al. reported improvements in gait speed of 10 mW since 1 h to 24 h post-CSF-TT [12]. On the contrary, many other studies recommend examining patients performances since the second day after the CSF-TT [17, 19, 20, 24]. Recently, Schniepp et al. examined 24 iNPH patients employing sequential recordings of gait velocity from 1 h to 72 h [25]. The maximal increase was observed in single-tasking after 24 h to 48 h, whereas in dual-tasking after 48 h to 72 h. The low negative predictive value in screening patients for shunting of CSF-TT (37%) might also depend on post evaluations not acquired on the appropriate time point [25].

The aim of this paper was threefold: i) to determine the contribution of gait analysis data obtained using IMUs and clinical scales on assessing pre vs. post-CSF-TT modifications in motor performance, ii) to determine the influence of the association between iNPH and vascular encephalopathy on CSF-TT outcomes, and iii) to evaluate whether CSF-TT effect on gait performance is more relevant after 24 or 72 h.

## Methods

### Participants

This observational prospective study was conducted between May 2015 to May 2018 in the Institute of Neurological Sciences (IRCCS) of Bologna, a national referral neurological and neurosurgical inpatient facility. Population eligible for inclusion in the study consisted of subjects: i) aged over 50 years old, ii) presenting at least one of the symptoms of the iNPH clinical triad [4], and iii) able to give verbal and written informed consent. Exclusion criteria were: i) the presence of severe psychiatric disease or physical illness, and ii) addiction to drugs. Patients eligible were admitted for investigation of iNPH by referral from neurologists, geriatricians, neurosurgeons and general practitioners and scheduled for a 3-Tesla-MRI brain scan. Patients with a clinical history possibly causing ventricular dilation, such as subarachnoid hemorrhage, meningitis, head injury, congenital hydrocephalus or aqueductal stenosis, were excluded. The diagnosis was assigned after reviewing: i) clinical data, ii) neuroimages, iii) neuropsychological information, and iv) blood and CSF composition tests during a consensus case conference comprising neurologists, neurosurgeons, neuropsychologists, neuroradiologists, physiatrists and nurses of the Institute of Neurological Sciences (IRCCS) of Bologna [26].

White matter changes were quantified on neuroimages with the Age-Related White Matters Changes (ARWMC) scale [27]. In this scale, the frontal, the parieto-occipital, the temporal, the infratentorial and the basal ganglia areas of right and left hemisphere are individually rated with a score ranging from 0 to 3 according to the number and degree of confluence of lesions. iNPH patients were classified in pure hydrocephalus (p-iNPH) or hydrocephalus with important vascular encephalopathy (v-iNPH) in case the overall score of ARWMC was respectively below or above 10. Considering the absence in the literature of a validated cut-off value, the one here proposed was established based on the expert opinion of the neuroradiologist.

This study was approved by the local ethics committee of the health service of Bologna, reference CE 14131 23/02/2015, and was conducted in agreement with principles of good clinical practice. All participants gave their written consent to participation according to the declaration of Helsinki.

### Protocol

The CSF-TT consisted in the removal of 30–40 ml of CSF using a 20-gauge spinal needle in lateral supine position.

Gait was assessed by TUG being the standard motor test in studies involving iNPH patients to assess balance and mobility [28], and by 10 mW extended to the distance of 18 m (18 mW), as proposed by Kahlon et al. [29]. During TUG patients raised from a chair with armrests, walked 3 m forward, turned 180° around a traffic cone, walked 3 m backward and sat back on the same chair. During 18 mW patients were instructed to walk on a straight line at a self-selected pace along a large and empty corridor 30 m long. Both tests were repeated three times in order to filter out the effect due to habituation or lack of attention. Gait was quantitatively assessed by TUG pre- (baseline), 24 h- (T24 h) and 72 h (T72 h) post-CSF-TT and by 18 mW at baseline and T24 h. In order to better infer the performance modification along the three time points, on the firsts 15 patients (10 p-iNPH and 5 v-iNPH) the 18 mW was also acquired at T72 h.

The TUG and 18 mW were instrumented using mGAIT (mHealth Technologies, Italy). In particular, three inertial sensors equipped with a triaxial accelerometer (full scale set at ±8 g) and a triaxial gyroscope (full scale set at ±1000°/s) and with a sampling rate of 100 Hz were wore two on the shoes and one on the lower trunk [30]. The sensors connected via Bluetooth to an Android smartphone using an app which functioned as a portable processing platform. The app implemented ad-hoc algorithms to detect the gait cycle events and an extended Kalman filter with zero velocity updates to determine the spatial gait parameters using the same framework reported in Ferrari et al. [15]. Threshold values and conditional expressions of the algorithms were tuned based on data coming from pathological gait patterns of patients with major neurological conditions.

Gait data were processed in real-time. At the end of tests the app provided the following parameters for analysis: i) test total time, ii) gait speed, iii) stride length, iv) cadence, v) phases of the gait cycle (duration of single and double support expressed as percentage of gait cycle, stance and swing), vi) phase coordination index (PCI) as a measure of gait coordination [31]. The trunk acceleration signals recorded during the TUG were processed, using Matlab 2017b (Mathworks Inc., USA), similarly to Palmerini et al. [32]. In particular, the trunk inclination was obtained through an inverse pendulum model as the arcsin of the vertical component of the acceleration normalized to gravity and zeroed to the initial sitting position. The following parameters were determined: vii) sit-to-stand duration, viii) number of steps to turn, ix) stand-to-sit duration, and x) time taken to walk.

At baseline and T72 h the following clinical scales were fulfilled: 1) Grading Scale, 2) Tinetti, 3) Gait Status Scale. For Grading Scale and Gait Status Scale, higher scores indicate more impaired motor abilities. Tinetti consists of: i) a balance section (Tinetti Balance), assessing 9 items scored out of 16 points, ii) a gait section (Tinetti Gait), assessing 8 items scored out of 12 points, and iii) a combined score (Tinetti) of 28 points as a result of the sum of the previous two. Higher scores mean less impaired motor abilities.

### Statistical analysis

Following what observed by Hollman et al. [33], in order to carefully evaluate all the complimentary aspects of gait that contribute to a modification in motor performance as a result of the CSF-TT, three parameters were selected as main responsive variables: i) cadence, ii) stride length, and iii) test total time.

With the goal of reducing measurement error, averaging of repeated measurements is common practice when analyzing outcomes in clinical research. However this procedure also removes useful information [34]. Furthermore, without properly considering test repetitions at the same time point as repeated within-subject measurements, efficiency and statistical power worsen. As also recently recommended by an editorial of Gait and Posture by Prescott RJ [35], the use of a linear model with mixed effects on non-averaged data can conversely properly account for correlation between repeated measurements, keep the narrowest confidence intervals for clinical parameters, hold the greatest statistical efficiency [36], and flexibly model time effects [37].

In this study, linear models with mixed effects were implemented using backward elimination for each of the three main responsive variables using R (https://www.r-project.org/). Backward elimination is a stepwise procedure that consists in including all the candidate variables to fit the statistical model and that, at each step, deletes the regressor (if any) whose loss gives most statistically insignificant deterioration to the fit of the reduced model, until no further variables can be deleted without a statistically significant loss of fit. Eq. 1 reports the complete model from which started the analysis of spatio-temporal gait parameters as regressors to the three response variables:

*Equation 1: Complete linear model with mixed effects used to determine effect of CSF-TT on modification of motor performance during instrumented TUG and 18 mW*

$$ {PerformanceMeasure}_{irt}={\beta}_0+{\beta}_1{T}_i+{age}_i+{BMI}_i+{diagnosis}_i+{doubleSupportDuration}_{irt}+{PCI}_{irt}+{turningSteps}_{irt}+{sitToStand}_{irt}+{standToSit}_{irt}+{walkTime}_{irt}+{b_0}_{it}+{\varepsilon}_{irt}\equiv {\mu}_{it}+{b_0}_{it}+{\varepsilon}_{irt} $$

where *PerformanceMeasure*_*irt*_ is the value of cadence, stride length or total time for the *r*-th measurement made at time *t* (*t* = baseline, T24 h, T72 h) on the *i*-th subject; *β*_0_ is the constant term, *β*_1_*T*_*i*_ is the term that models the trend of *PerformanceMeasure* along the three time points *t*. This model also includes an occasional-specific random intercept for each subject *b*_0*it*_, that takes into account possible correlations among the three test repetitions on same *t*.

In order to evaluate the contribution of clinical scales in addition to gait analysis parameters, to the three models reported in Eq. 1, the scores of Gait Status Scale, Tinetti Balance, Tinetti Gait, Tinetti, and Grading Scale were added, and the ones relative to T24 h were removed (Eq. 2). Proceeding likewise for Eq. 1, final fitting of the regression models was carried with backward elimination.

*Equation 2: Complete linear model with mixed effects used to determine effect of CSF during instrumented TUG and 18 mW using spatio-temporal gait parameters and clinical scales scores as regressors*

$$ Perfo\mathrm{r}{manceMeasure}_{irt}={\beta}_0+{\beta}_1{T}_i+{age}_i+{BMI}_i+{diagnosis}_i+{doubleSupportDuration}_{irt}+{PCI}_{irt}+{turningSteps}_{irt}+{sitToStand}_{irt}+{standToSit}_{irt}+{walkTime}_{irt}+ Gait\ {Status\ Scale}_{it}+{Tinetti\ Balance}_{it}+{Tinetti\ Gait}_{it}+{Tinetti}_{it}+{Grading\ Scale}_{it}+{b_0}_{it}+{\varepsilon}_{irt}\equiv {\mu}_{it}+{b_0}_{it}+{\varepsilon}_{irt} $$

Subscripts apply as in Eq. 1.

Finally, with the aim of analyzing the behavior of clinical rating scales alone with respect to responsive variables, a linear mixed model was realized using scores of clinical scales as the only predictors (Eq. 3).

*Equation 3: Complete linear model with mixed effects used to determine effect of CSF-TT using clinical scales scores as regressors*

$$ {PerformanceMeasure}_{irt}={\beta}_0+{\beta}_1{T}_i+{age}_i+{BMI}_i+{diagnosis}_i+ Gait\ {Status\ Scale}_{it}+{Tinetti\ Balance}_{it}+{Tinetti\ Gait}_{it}+{Tinetti}_{it}+{Grading\ Scale}_{it}+{\varepsilon}_{irt}\equiv {\mu}_{it}+{\varepsilon}_{irt} $$

Subscripts apply as in Eq. 1.

The contribution of each term on the right side of the three former equations, in the attempt to explain the variance of the three responsive variables on the left side, has been processed by the linear mixed models and eliminated just in case the deterioration to the fit of the reduced model was statistically insignificant.

## Results

### Participant flow

Among the 151 persons with suspicion of iNPH referred at IRCCS Institute, 76 patients received a diagnosis of iNPH. Fifty-six were p-iNPH forms whereas 20 were v-iNPH. Table 1 reports demographical, clinical and neurological characteristics of the iNPH sample and the number of motor test acquisitions.

**Table 1:** Baseline demographic, clinical and neurological characteristics of subjects and number of motor test acquisitions

	Total	p-iNPH	v-iNPH
Demographic			
Subjects	76	56	20
Males (%)	43 (57)	31 (55)	12 (60)
Mean age at disease onset (st. Dev)	72 (5.7)	71 (6.2)	73 (3.3)
Mean age at evaluation (st. Dev)	75 (4.7)	74 (5.1)	77 (3.0)
Disease duration < 12 months	21	18	3
Disease duration > 12 months	55	38	17
BMI, [kg/m^2^] (st. Dev)	27 (4.1)	28 (3.9)	25 (4.1)
Clinical profile			
Symptoms at first evaluation			
1, subjects (%)	6 (8)	6 (11)	0
2, subjects (%)	18 (24)	13 (23)	5 (25)
3, subjects (%)	52 (68)	37 (66)	15 (75)
gait disorders, subjects (%)	76 (100)	56 (100)	20 (100)
urinary dysfunctions, subjects (%)	63 (83)	47 (84)	16 (80)
cognitive impairments, subjects (%)	58 (76)	39 (70)	19 (95)
Number of falls in the last 6 months			
0, (%)	17 (22)	12 (21)	5 (25)
1, (%)	15 (20)	12 (21)	3 (15)
≥ 2, (%)	44 (58)	32 (57)	12 (60)
Mean ARWMC (st. Dev)	7.2 (4.6)	5.0 (2.7)	13.7 (2.3)
Motor tests			
TUG total trials acquired	585	405	180
TUG discarded (incomplete acquisitions)	31 (5%)	22 (5%)	9 (5%)
18 mW total trials acquired	405	300	135
18 mW discarded (incomplete acquisitions)	32 (8%)	21 (7%)	11 (8%)

*BMI* Body mass index*; ARWMC* Age-Related White Matters Changes

Patients unable to walk at baseline were excluded from the following analyses. Final dataset consisted of 45 p-iNPH (mean age (standard deviation): 73.9 (5.0) years; BMI: 28.1 (3.8) kg/m^2^) and 20 v-iNPH (mean age (standard deviation): 76.7 (3.1) years; BMI: 25.1 (4.0) kg/m^2^). These groups differed significantly in terms of mean age (t-test, *p* < 0.01) and mean BMI (t-test, *p* < 0.01); age and BMI were therefore included in the regression model to study their possible differential effect on performance (Eq. 1).

### Instrumental gait analysis of TUG and 18 mW

**Fig. 1:**
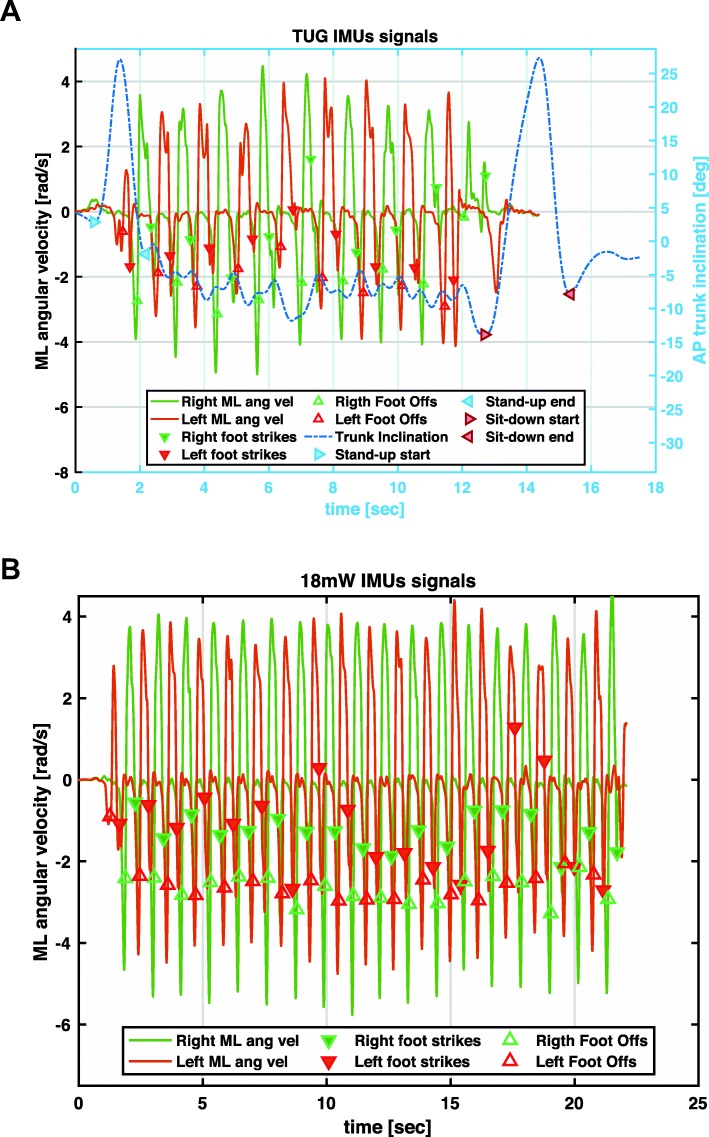
Typical IMUs recording for a) TUG and b) 18 mW of a p-iNPH subject acquired at T24 h. Heel strikes (downward triangles) and foot offs (upward triangles) are superimposed on top of shoes medio-lateral angular velocities (right in green and left in red); as well, starts (rightward triangles) and ends (leftward triangles) of sit-to-stand and stand-to-sit phases are shown (when present) on the antero-posterior inclination (in blue) recorded from the sensor placed on lower back

**Fig. 2:**
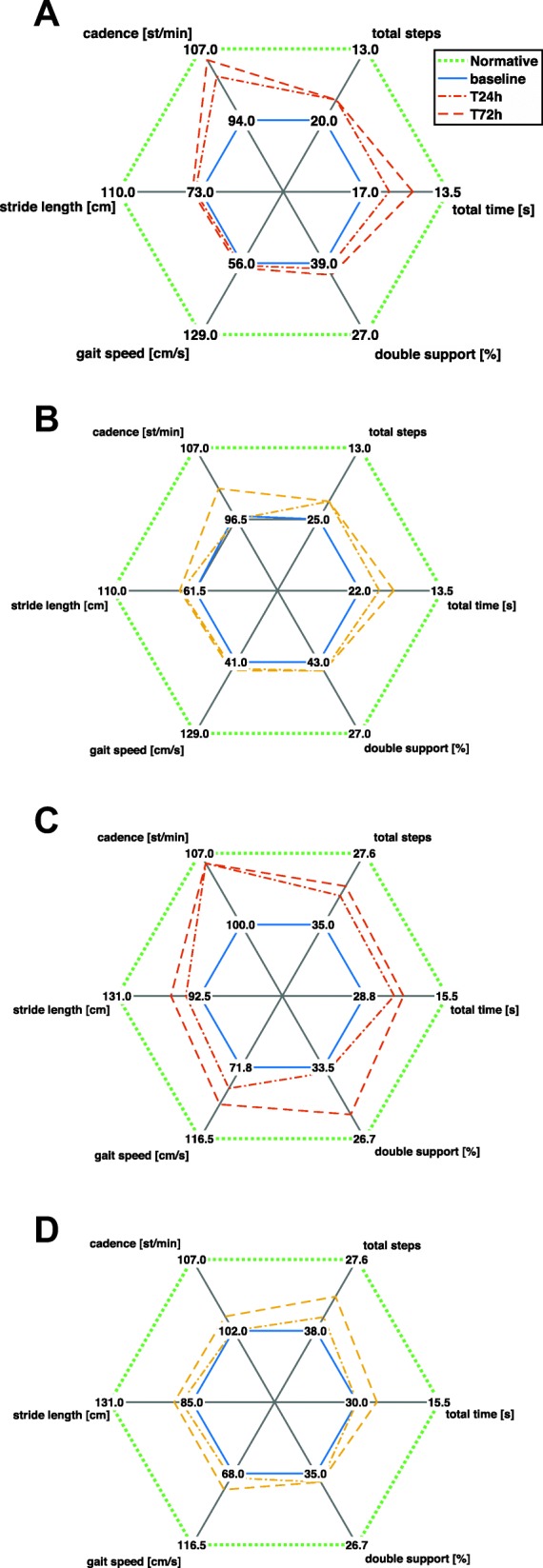
Radar plots of the progression of median values of cadence, stride length, total time, total steps, double support, and gait speed for **a** p-iNPH on TUG, **b** v-iNPH on TUG, **c** p-iNPH on 18 mW and **d** v-iNPH on 18 mW from baseline to T72 h. Distances are normalized with respect to baseline (blue lines) and normative values (green dot lines) derived for TUG from [18, 38]; for 18 mW from [33]. Red plots refer to the p-iNPH group, yellow plots to the v-iNPH group. Cadence is expressed in steps per minute

**Fig. 3:**
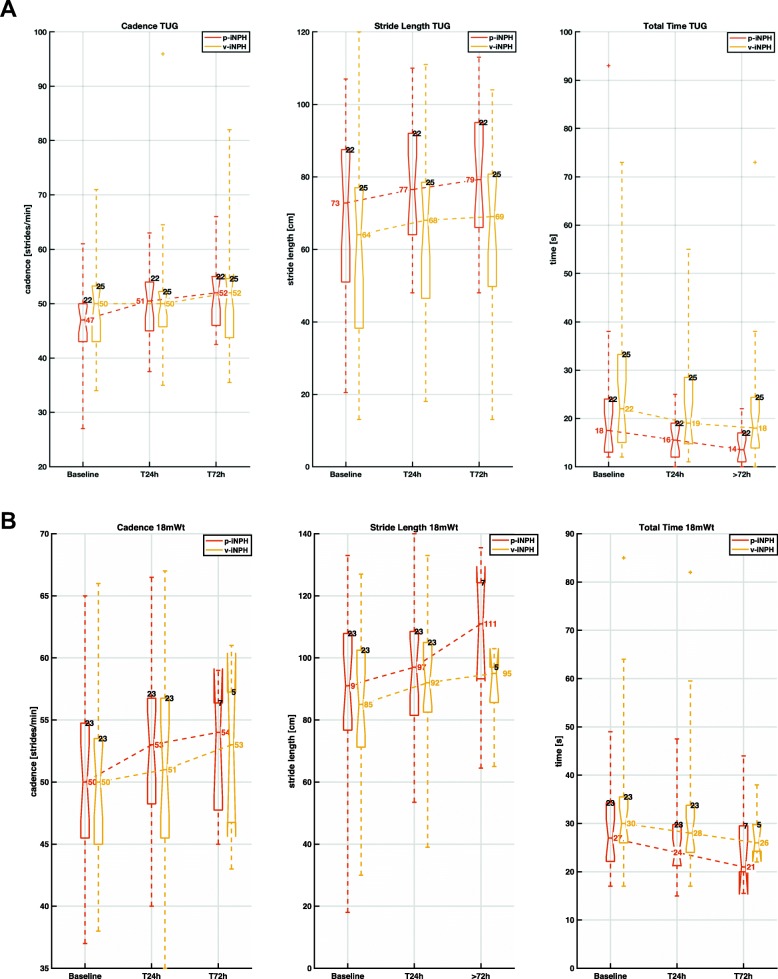
Box plot of all values obtained for responsive variables cadence, stride length and total time along the three time points baseline, T24 h and T72 h, and the two motor tests **a** TUG, and **b** 18 mW. Red boxes refer to the p-iNPH group (numbers in red report the median value), yellow boxes to the v-iNPH group (numbers in yellow report the median value). A single median value for the three repetitions was considered. Numbers in black represent the number of patients who successfully completed the tests

A total of 585 trials were acquired using mGAIT. Few of these trials were not completed either because patients lost focus on the task to accomplish or because they were too demanding from the physical point of view and were excluded from following analysis (Table 1). Three hundred eighty-three TUG and 279 18 mW trials for p-iNPH patients plus 171 TUG and 124 18 mW trials for v-iNPH patients were finally considered for the statistical analysis. Figure 1 shows a typical IMUs recording for TUG and 18 mW relative to a p-iNPH subject acquired at T24 h. In particular, heel strikes and foot offs are superimposed on top of shoes medio-lateral angular velocities; as well starts and ends of sit-to-stand and stand-to-sit phases on top of trunk antero-posterior acceleration. Figure 2 shows in a radar chart the progression, for the two groups and the two motor tests, of the median cadence, stride length, total time, total steps, double support, and gait speed along the three time points (baseline, T24 h and T72 h) using normative data as reference. Figure 3 shows more in detail via box plots all values obtained for gait speed across the two groups, the three time points, and the two motor tests.

Table 2 reports the final statistical model, after regressor selection via backward elimination. Comparing results at baseline vs. T24 h and T72 h, the TUG disclosed a statistically significant improvement for cadence both at T24 h (*p* < 0.01) and T72 h (*p* < 0.001), for total time just at T72 h (*p* < 0.05), whereas stride length modifications were statistically not relevant. The 18 mW revealed both for stride length and cadence an improvement at T24 h (*p* < 0.05 and *p* < 0.001, respectively) and at T72 h (*p* < 0.05 in both cases), however total time never showed the time effect as significant. Belonging to p-iNPH or v-iNPH did not provide a relevant contribution to explain any of the outcome measures. However, values obtained disclosed on average a decreased performance of v-iNPH subjects compared to p-iNPH subjects, with a trend towards significance in 18 mW where v-iNPH showed on average an increase of 2 s on test total time.

**Table 2:** Results of the three linear models with mixed effects built on the responsive variables stride length, cadence and total time (Eq. 1) from gait analysis data of TUG and 18 mW at baseline, T24 h and T72 h

	TUG			18 mW		
		Value	*p*-value		Value	*p*-value
Stride length [cm]	(Intercept)	140.3	0.000	(Intercept)	157.2	0.000
	T24 h	−0.1	0.878	T24 h	1.8	0.023 *
	T72 h	1.4	0.113	T72 h	3.1	0.027 *
	v-iNPH vs p-iNPH	−3.5	0.237	v-iNPH vs p-iNPH	−5.5	0.164
	double support duration	−1.6	0.000 ***	double support duration	−1.9	0.000 ***
	PCI	−0.02	0.024 *	PCI	−0.1	0.001 ***
	n. turning steps	−2.0	0.000 ***			
Cadence [strides/min]	(Intercept)	58.8	0.000	(Intercept)	63.3	0.000
	T24 h	1.8	0.009 **	T24 h	1.5	0.000 ***
	T72 h	2.4	0.001 ***	T72 h	1.7	0.018 *
	v-iNPH vs p-iNPH	−0.6	0.774	v-iNPH vs p-iNPH	−0.7	0.654
	double support duration	−0.3	0.000 ***	double support duration	−0.4	0.000 ***
	PCI	−0.01	0.006 **	PCI	0.1	0.000 ***
	n. turning steps	0.4	0.015 *			
Total time [s]	(Intercept)	−10.5	0.000	(Intercept)	−10.8	0.001
	T24 h	−1.8	0.190	T24 h	−2.9	0.108
	T72 h	−2.8	0.031 *	T72 h	−2.7	0.160
	v-iNPH vs p-iNPH	0.4	0.677	v-iNPH vs p-iNPH	2.0	0.137
	double support duration	0.6	0.000 ***	double support duration	1.2	0.000 ***
	PCI	0.1	0.000 ***	PCI	0.2	0.000 ***
	n. turning steps	1.7	0.000 ***			
	Sit-down duration	0.5	0.000 ***			
	Stand-up duration	0.5	0.005 **			

*T24 h and T72 h* post 24 and 72 h the CSF tap test;

*v-iNPH vs p-iNPH* pure iNPH group vs iNPH with vascular encephalopathy comorbidity group;

*PCI* phase coordination index;

Values of stride length are expressed in cm, of cadence in strides/min, of total time in s;

Significance codes: ‘***’ p < 0.001, ‘**’ *p* < 0.01, ‘*’ *p* < 0.05

The change in stride length, cadence and total time measured with TUG, other than pre vs. post comparison, was mostly predicted by the reduction of the duration of double support, the number of turning steps, and the value of PCI. In particular, double support duration showed a high statistical significance (*p* < 0.001) across the two motor tests, where to its unitary increment corresponded an average decrease of stride length (of 1.6 cm in TUG and 1.9 cm in 18 mW), and cadence (of 0.3 in TUG and 0.4 strides/min in 18 mW) and, on average, an increase of total time (of 0.6 s in TUG and 1.2 s in 18 mW). The time to complete TUG was also explained by duration of sit-to-stand and stand-to-sit phases. As well, the 18 mW was predicted both by the duration of double support and PCI.

In order to verify the homoscedasticity of random effects, the Levene test was performed on model residuals and reported in Table 3. In particular, the variability among subjects excluding the time effect, the variability among time points including all subjects’ trials, and the intersection between these latter two were considered.

**Table 3:** Levene’s Test for homogeneity of variance on residuals of statistical model of Eq. 1

	TUG			18 mW		
	Stride length *p*-value	Cadence *p*-value	Total time *p*-value	Stride length *p*-value	Cadence *p*-value	Total time *p*-value
i	0.001***	0.000***	0.000***	0.002**	0.003**	0.001***
t	0.913	0.374	0.835	0.016*	0.536	0.049*
i*t	0.999	0.400	0.017*	0.993	0.829	0.787

*i* variability among subjects*;*

*t* variability among time points baseline, T24 h and T72 h*;*

i*t: variability among subjects and time points*;*

Significance codes: ‘***’ *p* < 0.001, ‘**’ *p* < 0.01, ‘*’ *p* < 0.05

The homoscedasticity of random effects is never present (*p* < 0.003) when the inter-subject variability alone is considered (Table 3), showing how patients performances differ considerably among themselves and along time points. On the contrary, during TUG along time homoscedasticity is always present (*p* > 0.3) showing how overall variability at baseline, T24 h and T72 h did not differ. Also on 18 mW homoscedasticity is present along time for cadence but not for stride length and total time, testifying how from baseline to T72 h the variability changed. Finally, considering the time-subject interaction the homoscedasticity is always present both for TUG and 18 mW except for the outcome total time in TUG (*p* < 0.05).

### Clinical scales

The clinical scales were fulfilled on 56 p-INPH and 20 v-iNPH patients. However, in few cases patients were not able to perform clinical test tasks; Table 4 reports mean, standard deviation and number of patients able to execute the tests. At baseline, v-iNPH patients presented on average higher mean values for Gait Status Scale when compared to p-iNPH, 6.4 vs. 5.5 respectively, but without reaching statistical significance. Tinetti Balance, Tinetti gait, Tinetti, and Grading Scale also did not show relevant differences between groups (Table 4).

**Table 4:** Mean scores of clinical scales

	p-iNPH		v-iNPH	
Mean scores	Baseline	T72 h	Baseline	T72 h
Gait Status Scale (st. Dev) [subjects]	5.5 (3.8) [54]	4.3 (3.5) [53]	6.4 (3.3) [19]	4.3 (2.7) [18]
Tinetti Balance	12.0 (3.7) [54]	13.8 (2.5) [52]	11.7 (2.9) [19]	13.1 (2.6) [18]
Tinetti Gait	7.0 (3.4) [54]	8.8 (2.6) [52]	6.8 (3.0) [19]	8.6 (2.5) [18]
Tinetti	18.9 (6.6) [54]	22.4 (4.7) [52]	18.6 (5.4) [19]	21.7 (4.6) [18]
Grading Scale	5.9 (2.4) [56]	4.5 (2.1) [53]	5.9 (2.3) [19]	5.2 (2.7) [18]

*st. Dev* standard deviation;

[subjects]: number of subjects able to execute the tasks of the clinical scale;

*T72 h* post 72 h the CSF tap test;

*p-iNPH* pure iNPH group;

*v-iNPH* iNPH with vascular encephalopathy comorbidity group

Table 5 reports final fitting of the regression models of the contribution of clinical scales in addition to gait analysis parameters as specified in Eq. 2.

**Table 5:** Results of the statistical analysis on the outcome measures stride length, cadence and total time considering as predictors both the instrumental gait analysis parameters and the scores of clinical scales (model defined in Eq. 2)

	TUG			18 mW		
		Value	*p*-value		Value	*p*-value
Stride length [cm]	(Intercept)	135.4	0.000	(Intercept)	164.3	0.000
	T72 h	−1.9	0.064	T72 h	2.9	0.071
	v-iNPH vs p-iNPH	−2.0	0.414	v-iNPH vs p-iNPH	−3.1	0.307
	double support duration	−1.6	0.000 ***	double support duration	−1.9	0.000 ***
	n. turning steps	−2.9	0.000 ***	PCI	−0.1	0.035 *
	Tinetti Gait	1.2	0.004 **	Tinetti Gait	2.8	0.000 ***
	Gait Status Scale	−1.0	0.010 **	Gait Status Scale	−2.0	0.004 *
				Tinetti Balance	−1.6	0.043 *
Cadence [strides/min]	(Intercept)	59.5	0.000	(Intercept)	63.4	0.000
	T72 h	3.0	0.000***	T72 h	1.6	0.070
	v-iNPH vs p-iNPH	−0.9	0.670	v-iNPH vs p-iNPH	0.1	0.944
	double support duration	−0.4	0.000 ***	double support duration	−0.4	0.000 ***
	n. turning steps	0.7	0.002 **	PCI	0.1	0.001 ***
	Gait Status Scale	0.6	0.004 **			
Total time [s]	(Intercept)	2.5	0.549	(Intercept)	13.9	0.032
	T72 h	−1.6	0.156	T72 h	−1.7	0.446
	v-iNPH vs p-iNPH	0.3	0.757	v-iNPH vs p-iNPH	1.2	0.482
	double support duration	0.7	0.000 ***	double support duration	0.8	0.000 ***
	PCI	0.03	0.000 ***	PCI	0.2	0.000 ***
	Sit-down duration	0.6	0.000 ***	Tinetti Gait	−1.4	0.001 ***
	Tinetti Balance	−0.9	0.000 ***			

*T72 h* post 72 h the CSF tap test;

*v-iNPH* vs *p-iNPH* pure iNPH group vs iNPH with vascular encephalopathy comorbidity group

*PCI* phase coordination index

Significance codes: ‘***’ p < 0.001, ‘**’ *p* < 0.01, ‘*’ *p* < 0.05

Considering the results of TUG, reported in Table 5, compared to the ones relative to the model of Eq. 1, PCI remained significant just in the prediction of total time. Both Tinetti Gait and Gait Status Scale were not eliminated from the model fitting of stride length and in correspondence to their unitary increment, stride length increased respectively of 1.2 cm and decreased of 1 cm, on average. Grading Scale, as well, was considered as an explanatory variable in prediction of cadence with an average increase of 0.6 strides/min per its unitary increment. Finally, Tinetti Balance was the only clinical scale remaining in the prediction of total time, providing a contribution more relevant with respect to sit-to-stand time and turning steps. TUG total time decreased on average of 0.9 s per single increment of Tinetti Balance.

When considering cadence in 18 mW results (Table 5), all scales gave statistically insignificant deterioration to the fit of the reduced model and were therefore eliminated. Tinetti gait was the only scale not eliminated for total time, which was reduced of 1.4 s, on average, per its unitary increment. Tinetti Gait, Grading Scale and Tinetti Balance gave their relevant contribution to explain stride length variability. In correspondence of a unitary increment of Tinetti Gait, Grading Scale and Tinetti Balance, stride length respectively increased of 2.8 cm and decreased of 1.6 cm and 2 cm, on average. As in previous model, the homoscedasticity of random effects was verified by means of the Levene test on model residuals (Table 6).

**Table 6:** Levene’s Test for homogeneity of variance on residuals of statistical model of Eq. 2

	TUG			18 mW		
	Stride Length	Cadence	Total time	Stride Length	Cadence	Total time
i	0.000***	0.000***	0.000***	0.344	0.341	0.141
t	0.847	0.592	0.538	0.008**	0.495	0.013*
i*t	0.989	0.146	0.001***	0.882	0.771	0.733

*i* i-th subject

*t* time point: baseline, T24 h or T72 h

Significance codes: ‘***’ p < 0.001, ‘**’ *p* < 0.01, ‘*’ *p* < 0.05

Same considerations as for the model described by Eq. 1 can be applied here. In the interaction time-subject the homoscedasticity is always present both for TUG and 18 mW except for the outcome total time in TUG (*p* < 0.001).

The contribution of clinical rating scales alone to explain responsive variables, as detailed in Eq. 3, is reported in Table 7 During TUG stride length was predicted with statistical significance by Gait Status Scale, with a unitary increase of this scale producing a stride 1.7 cm shorter, on average. For 18 mW, other than Gait Status Scale shortening on average the stride of 2.3 cm per unitary increment, Tinetti Gait was also not eliminated producing a stride 5.9 cm longer per unitary increment, on average. Grading scale significantly contributed to explain total time and cadence variability during TUG, with its unitary increment determining respectively an increase of 1.8 s and a decrease of 0.9 strides/min, on average. Time effect resulted also statistically relevant for cadence both during TUG and 18 mW (Table 7).

**Table 7:** Results of the statistical analysis on the outcome measures stride length, cadence and total time considering the scores of clinical scales as the only predictors (model defined in Eq. 3)

	TUG			18 mW		
		Value	*p*-value		Value	*p*-value
Stride length [cm]	(Intercept)	46.9	0.000	(Intercept)	85.6	0.000
	T72 h	−2.3	0.206	T72 h	4.0	0.130
	Tinetti Gait	2.4	0.209	Tinetti Gait	5.9	0.002**
	Tinetti Balance	1.0	0.583	Tinetti Balance	−1.6	0.392
	Tinetti	0.2	0.902	Tinetti	−0.4	0.808
	Gait Status Scale	−1.7	0.013*	Gait Status Scale	−2.3	0.022*
	Grading Scale	−1.4	0.060	Grading Scale	−0.7	0.471
Cadence [strides/min]	(Intercept)	51.7	0.000	(Intercept)	61.0	0.000
	T72 h	2.8	0.002**	T72 h	2.5	0.023*
	Tinetti Gait	−0.3	0.733	Tinetti Gait	−0.2	0.838
	Tinetti Balance	0.3	0.702	Tinetti Balance	−0.2	0.825
	Tinetti	−0.1	0.883	Tinetti	−0.1	0.845
	Gait Status Scale	0.4	0.231	Gait Status Scale	−0.5	0.247
	Grading Scale	−0.9	0.021*	Grading Scale	−0.4	0.398
Total time [s]	(Intercept)	30.0	0.000	(Intercept)	38.7	0.006
	T72 h	−1.6	0.248	T72 h	−2.7	0.338
	Tinetti Gait	0.01	0.988	Tinetti Gait	−2.2	0.096
	Tinetti Balance	−0.6	0.482	Tinetti Balance	1.1	0.338
	Tinetti	−0.3	0.670	Tinetti	−0.5	0.598
	Gait Status Scale	0.5	0.156	Gait Status Scale	0.6	0.391
	Grading Scale	0.8	0.013*	Grading Scale	0.8	0.359

*T72 h* post 72 h the CSF tap test;

Significance codes: ‘**’ p < 0.01, ‘*’ *p* < 0.05

## Discussion

This observational cohort study is one of the largest monocentric studies carried out so far on idiopathic normal pressure hydrocephalus, a complex and often misdiagnosed syndrome. In this study, out of 151 patients with suspected hydrocephalus from brain imaging and at least one symptom of the classical triad, 76 were diagnosed as iNPH by a clinical multidisciplinary team following a standardized protocol.

Motor performance was measured using inertial sensors which allowed to instrument the TUG and the 18 mW tests without affecting the clinical routine. More so, IMUs allowed to measure gait parameters such as the duration of the double support phase and the PCI that are directly related to typical disturbances of iNPH as the presence of magnetic gait and of apraxia. Cadence, stride length and total time, being three main complimentary components of locomotion, were selected as response variables of statistical models implemented to assess modifications caused by CSF-TT, hence to establish and quantify its effect on symptoms improvement. The choice to use a 18 mW allowed the stress the locomotor abilities of patients, but the use of the more standard 10 mW would have allowed a broader opportunity of comparisons with the literature and in future studies might be preferred.

Comparing gait analysis results obtained in the TUG test at baseline and post-CSF-TT, on average, as showed in Fig. 3a, patients increased their cadence and reduced the time required to complete the test (Table 2). Conversely, stride length did not change, probably due to the nature of TUG that limits free walking to just 3 m. On the other hand, in the 18 mW test stride length and cadence significantly improved, whereas the change in total time, even showing a reduction of more than 2 s on average (Fig. 3b, Table 2), did not reach statistical significance. This latter result might be partially explained by the fact that at T72 h just 15 patients performed the 18 mW causing a reduced statistical power compared to TUG. More so, this might suggest that the performance of iNPH patients is subject to modifications not necessarily involving gross motor skills, but aspects related to the quality of motor control such as the onset of apraxia. In line with this interpretation, double support duration and PCI were the two regressors most influent for explaining total time revealing how moving from baseline to T72 h patients exhibited a gait less magnetic and apraxic, and more coordinated and fluent.

Considering clinical scores analyzed in addition to instrumental measures as in Eq. 2 (results in Table 5), from baseline to T72 h patients increased their cadence significantly in TUG. As well, concerning clinical scores analyzed by themselves as in Eq. 3 (results Table 7), the cadence is increased both in TUG and 18 mW. Time effect in the explanation of the variability of performance measures is therefore attenuated by the addition of clinical scores to statistical models. When looking at the individual contribution of each scale, there was not a single scale fitting well in all statistical models. Tinetti Gait and Gait Status Scale were both explanatory variables in prediction of stride length both for TUG and 18 mW in the model including quantitative measures (Table 5), but only for 18 mW in the model with clinical scales alone (Table 7), revealing their ability to address stride length variations besides instrumental data. Tinetti Gait was the only scale remaining significant in explaining the variability of total time for 18 mW (Table 5), hence showing its affinity to gait speed during an unconstrained walk. Tinetti Balance, along with gait analysis data, Tinetti Gait and Gait Status Scale were significant for predicting stride length in 18 mW (Table 5). More so, Tinetti Balance was the only scale predictive of TUG total time (Table 5), probably reflecting its ability to address those components of motor organization related to the pattern adopted to find and maintain balance during the sit-to-stand and stand-to-sit postural transfers. For TUG, in the model including instrumental data, to a unitary increment of Gait Status Scale, theoretically corresponding to a worsening of patients’ motor abilities, corresponded a significant increase of cadence of 0.6 steps/min as well as a 1 cm reduction of stride length, on average. These results, though partially controversial, probably reveal the presence of shuffling gait for patients with higher values of Gait Status Scale. In other words, with the increase of values of Gait Status Scale the performance deteriorates in terms of reduced stride length and, counterintuitively, increased cadence, which clinically can be associated to the presence of a shuffling gait.

Tinetti Gait, Tinetti Balance and Gait Status Scale in TUG provided a contribution more relevant with respect to stand-up time and turning steps in total time, and to PCI in stride length and cadence. This result can be probably ascribed to their capability of assessing a broad spectrum of top-down components of the motor repertoire necessary to execute the TUG. On the contrary, Tinetti global score and Grading Scale when analyzed with instrumental data were never selected for model fitting, revealing their inability to unveil undisclosed aspects of performance. Overall, the fact that some clinical scales in the statistical model of Eq. 2 provided a contribution more relevant with respect to gait analysis parameters also suggests that other instrumental data, obtained from inertial sensors or other devices, might be considered in order to fully describe pre vs post-CSF-TT modifications of motor performance just in terms of objective data.

Grading Scale on the other hand, in the model with clinical scales alone, was the only scale found significant for cadence and total time in TUG, demonstrating its ability to explain the majority of the variability of these two outcome measures to the detriment of other clinical scales.

When analysing the variance of statistical model residuals relative to the interaction between time and subjects, the homoscedasticity is always present except for the total time in TUG (Table 3 and Table 6). This latter finding possibly indicates that a factor relevant for the determination of the parameter total time has not been taken into account into the statistical model. Based on clinical observations, this factor might have been the cognitive ability to correctly implement the test without hesitations, that was frequently compromised at baseline but often recovered at T72 h. Indeed, patients at baseline sometimes got lost during test execution, for example continuing to walk straight instead of turning and returning to the chair. In these cases, the TUG was disclosing not only the locomotor abilities but also the cognitive functioning of patients. By inserting the scores of a scale such as the Mini Mental State Examination [39] in the statistical model of total time as a fixed effect, could effectively account for the presence and severity of cognitive impairment and its role in the variation of the motor performance. Further research aimed at confirming this hypothesis should be carried out.

v-iNPH patients showed on average a reduced performance compared to p-iNPH in all the different observations but without reaching the statistical significance. Contrary to authors expectations, important vascular encephalopathy present in persons with v-iNPH was not a factor causing a different post-CSF-TT course. This result might be partially explained by the fact that the high individual variability modeled with the random intercept captured only part of the variability ascribed to the two iNPH groups. On the other hand, this result suggests that vascular encephalopathy is not a comorbidity limiting the improvement of gait following CSF-TT. In the present study, the ARWMC was dichotomized in order to ease the clinical interpretation of the results. However, including this variable into the statistical models themselves as fixed effect might increase the variance explained and turn out to be a significant predictor of the responsive variables.

When comparing different post-CSF-TT assessment times, the better performances were recorded 72 h after the CSF-TT. This finding is clearly visible in the radar plots of Fig. 2 where the hexagon relative to median values of spatio-temporal gait parameters recorder at T72 h is always including, meaning better performance, the one relative to T24 h. These results are in agreement with those reported by Schniepp et al. but in contrast to other studies [10, 22], and in particular with the work of Virhammar et al. that proposed to make the post-CSF-TT evaluation any time within the first 24 h [12]. These findings also suggest that previous studies investigating motor improvements in early time points could have underestimated the CSF-TT response.

## Conclusions

Inertial sensors used to instrument motor tests on iNPH patients allowed to assess changes on performances due to CSF-TT without affecting the clinical routine and unveiling aspects of locomotion and postural transfers not accessible simply using clinical observations or clinical scales. On average, patients improved the performance from baseline to post-CSF-TT particularly increasing cadence in 18 mW and TUG and stride length in 18 mW. More so, the use of data from inertial sensors and mixed effects models allowed to show how the improvement was significant especially in those gait parameters reflecting a decrease in apraxia such as the reduction of double support duration and the increase of coordination.

Tinetti Gait, Tinetti Balance and Gait Status Scale were the scales able, better than others, to address part of the variability of response variables not explained by sensor data. Additionally, these scales in TUG provided a contribution more relevant with respect to stand-up time and turning steps in total time, and to PCI in stride length and cadence, showing the capability to assess components of the motor repertoire not appropriately addressed by gait parameters. On the other hand, when considering clinical scales alone, Grading Scale was the scale with highest affinity in explaining TUG total time and cadence.

v-iNPH patients compared to p-iNPH showed worst performances in all evaluations but without reaching statistical significance. This comorbidity was not a factor causing a different post-CSF-TT course by limiting gait improvement.

Maximal increase in outcome measures occurred the third day after the CSF-TT, revealing how this interval should be the considered as the best spot to assess CSF-TT efficacy in iNPH.

In conclusion, the results obtained in this study suggest that it is possible to improve the assessment of effectiveness of the CSF-TT by including the instrumented evaluation of spatio-temporal gait parameters and setting the time for post-CSF-TT evaluation at 72 h.

## Data Availability

Please contact corresponding author.

## Abbreviations

18 mW: 10 m walking test (10 mW) extended to the distance of 18 m
ARWMC: Age-Related White Matters Changes scale
CSF: Cerebrospinal fluid
IMUs: Inertial measurement units
iNPH: Idiopathic normal pressure hydrocephalus.
PCI: Phase coordination index
p-iNPH: Pure iNPH group
TT: Tap-test
TUG: Timed-up and go test
v-iNPH: iNPH with vascular encephalopathy comorbidity group
